# Microbiologic Characteristics, Serologic Responses, and Clinical Manifestations in Severe Acute Respiratory Syndrome, Taiwan^1^

**DOI:** 10.3201/eid0909.030367

**Published:** 2003-09

**Authors:** Po-Ren Hsueh, Cheng-Hsiang Hsiao, Shiou-Hwei Yeh, Wei-Kung Wang, Pei-Jer Chen, Jin-Town Wang, Shan-Chwen Chang, Chuan-Liang Kao, Pan-Chyr Yang, The SARS Research Group of National Taiwan University College of Medicine and National Taiwan University Hospital

**Affiliations:** * National Taiwan University Hospital, National Taiwan University College of Medicine, Taipei, Taiwan; † National Health Research Institute, Taipei, Taiwan

## Abstract

The genome of one Taiwanese severe acute respiratory syndrome-associated coronavirus (SARS-CoV) strain (TW1) was 29,729 nt in length. Viral RNA may persist for some time in patients who seroconvert, and some patients may lack an antibody response (immunoglobulin G) to SARS-CoV >21 days after illness onset. An upsurge of antibody response was associated with the aggravation of respiratory failure.

In November 2002, cases of a life-threatening and highly contagious febrile respiratory illness of unknown cause were reported from Guangdong Province in southern China, followed by reports from Vietnam, Hong Kong, Singapore, Canada, the United States, and other countries (*1*–*4*). This illness was identified as a new clinical entity and designated as severe acute respiratory syndrome (SARS) in late February 2003. This disease has a high propensity to spread to healthcare workers and household members and may cause outbreaks in the community (*1*–*4*). Recent reports have demonstrated that a novel coronavirus, SARS-associated coronavirus (SARS-CoV), is associated with the pathogenesis of SARS (*5*–*7*). Laboratory diagnostic tests to analyze clinical specimens for SARS-CoV include reverse-transcriptase polymerase chain reaction (RT-PCR) specific for RNA and detection of specific antibody by using indirect fluorescence antibody and enzyme-linked immunosorbent assays (*8*,*9*). However, data on the timing and intensity of serologic responses after illness onset and the association of these responses with clinical manifestations of the disease are lacking.

In Taiwan, the first case of SARS occurred in a businessman working in Guangdong who was admitted to National Taiwan University Hospital (NTUH) on March 8, 2003. As of May 18, 2003, a total of 308 probable cases of SARS were reported by the Center for Disease Control, Department of Health, Taiwan (*10*).

## The Study

This study included seven Taiwanese patients, treated at the National Taiwan University Hospital from March 8 to May 3, 2003, whose illness met the recent Centers for Disease Control and Prevention (CDC) and World Health Organization (WHO) case definition for probable cases of SARS (*11*,*12*). The patients were 26–53 years of age, and six were men. The incubation period ranged from 2 to 12 days. Of the seven patients, four had recently returned from China: two patients (patients 1 and 7) from Guangdong Province and two (patients 5 and 6) from Beijing. In addition, two family members (patients 2 and 3), and one healthcare worker (patient 4) were from a cluster, which had household or healthcare contact with a SARS patient, and two patients (patient 5 and 6) were from another cluster, which had close contact with a SARS patient in an airplane.

All patients had fever (body temperature >38°C) and dry cough. Other symptoms included malaise (five patients), myalgia (five patients), and rigor (four patients). All but one patient (patient 7) had loose stools or diarrhea 2–10 days after febrile episodes, and five, including the four cluster A patients, had aggravating diarrhea 9–14 days after febrile episodes. The mean interval between onset of symptoms and hospitalization was 7.3 days (range 4–12 days).

Pneumonia developed in all seven patients, acute respiratory distress syndrome (ARDS) developed in four (patients 1, 2, 3, and 6), and ventilator support was given 10–12 days after the onset of illness. Pancytopenia compatible with hemophagocytosis syndrome developed in patient 2. Five patients (patients 2, 3, 4, 5, and 6) received ribavirin, intravenous corticosteroid (methylprednisolone, 2 mg/kg/d), and intravenous immunoglobulin (IVIG, 1 gm/kg/d for 2 days). Interstitial pneumonia developed in patient 7, who responded well to ribavirin and antibiotic treatment. All patients survived.

Urinary antigen detection for *S. pneumoniae* and *Legionella pneumophila* serogroup I was negative in all seven patients. Serum from patient 5 was positive for *Mycoplasma pneumoniae* immunoglobulin (Ig) M (enzyme-linked immunosorbent assay [ELISA]) antibody with a fourfold increase in complement fixation (CF) antibody titer in acute- (<1:40) and convalescent-phase sera (1:160). An elevated *Chlamydia* *pneumoniae* CF antibody (1:32) but negative reaction for *C. pneumoniae* IgM (ELISA) antibody was found in the acute-phase serum sample from patients 1 and 6 and in the acute- (1:32) and convalescent-phase serum (1:32) samples from patients 5 and 7. The antibody titers of acute- and convalescent-phase serum samples for *C. pneumoniae, C. trachomatis, C. psittaci,* and *L. pneumophila* in the other patients showed no significant increase. Five patients (patients 1, 2, 4, 5, and 6) had elevated CF antibody levels (≥1:16) against parainfluenzavirus 1, 2, or 3. Cultures for influenza virus, parainfluenzavirus, mumps, respiratory syncytial virus, adenovirus, enterovirus, herpes simplex virus, varicella-zoster virus, and cytomegalovirus were negative from various clinical samples of these patients.

Nucleic acid was extracted from the sputum and serum samples and the infected Vero E6 cells by using a viral RNA kit (QIAamp, Qiagen Inc., Valencia, CA). Reverse transcription polymerase chain reaction (RT-PCR) for SARS-CoV was performed with 3 sets of primers (IN-6 and IN-7; Cor-p-F1 and Cor-p-R2; and BNIinS and BNIAs) developed by CDC and WHO Network Laboratory. The RT-PCR products were analyzed, and the unique fragment was cloned and sequenced (*6*,*11*). RT-PCR test results for SARS-CoV were positive in oropharyngeal swabs from patients 6 and 7; sputum from patients 1, 2, 3, 4, and 5; and serum specimens from patients 1, 2, 3, 4, 5, and 7. Cultures of all oropharyngeal swabs and serum specimens were negative.

Cytopathic effects in the Vero E6 cells were first found between day 3 and day 4 after injection of serum specimens from patients 3 and 4. The initial cytopathic effect was focal**,** with cell rounding, and was followed by cell detachment. Similar cytopathic effects developed rapidly (between day 2 and day 3) after subculture.

**Figure 1:**
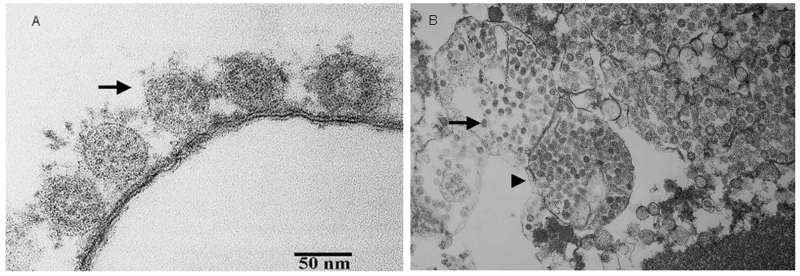
Thin-section electron micrograph of severe acute respiratory syndrome-associated coronavirus grown in Vero E6 cells. Panel A shows extracellular viral particles (arrow) lining the surface of the plasma membrane. Some spikes projecting from the envelope of the virus are seen. Panel B shows numerous spherical coronavirus particles (arrow) within dilated cytoplasmic vacuoles (arrowhead).

Ultra-thin sections were prepared for electron microscopy by fixing a washed infected Vero E6 cell pellet with 2.5% glutaraldehyde and embedding in Spurr’s resin. The SARS-CoV (range 60–80 nm in diameter) was identified by electron microscopy (Figure 1 A and B). RT-PCR from the infected Vero-E6 cells identified the same amplicon. Sequences of the amplicons from all patients were identical and were also identical to those from infected Vero E6 cells.

The genome of the SARS-CoV (TW1) (GenBank accession no., AY291451) strain from patient 3 was 29,729 nt in length. A comparison of TW1 sequences to the sequences described previously is summarized in the Table. The number of nucleotide differences between this TW1 isolate and the Urbani (AY278741), Tor-2 (AY274119), HKU-39848 (AY278491), and CUHK-W1 (AY278554) strains was 6, 3, 12, and 10, respectively.

**Table:** . Nucleotide base differences among the TW-1, TOR-2, HKU-39849, CUHK-W1, and the Urbani sequences of SARS-CoV^a^

SARS-associated coronavirus sequence							
Base	TW-1	TOR-2	HKU-39849	CUHK-W1	Urbani	A. a. change^b^	
						TW1/Urbani	
2,601	T	T	C	T	T	Val/Val	
3,165	G	A	A	A	A	Ser/Ser	
7,746	G	G	T	T	G	Pro/Pro	
7,919	C	C	C	C	T	Ala/Val	
9,404	T	T	C	C	T	Val/Ala	
9,479	T	T	C	C	T	Val/Ala	
16,622	C	C	C	C	T	Ala/Ala	
17,564	T	T	G	G	T	Asp/Glu	
17,846	C	C	T	T	C	Arg/Arg	
19,064	A	A	G	G	G	Glu/Glu	
21,721	G	G	A	A	G	Gly/Asp	
22,222	T	T	C	C	T	Ile/Thr	
23,220	T	G	T	T	T	Ser/Ala	
24,872	T	T	T	T	C	Leu/Leu	
25,298	G	A	G	G	G	Gly/Arg	
26867	T	T	T	T	C	Ser/Pro	
27,827	T	T	C	C	T	Cys/Arg	

^a^SARS, severe acute respiratory syndrome. ^b^Indicates a base difference resulting in an amino acid change.

IgG antibody to the SARS-CoV was detected by a standard indirect fluorescence antibody assay (IFA) with serial serum specimens from the seven patients. Spot slides for IFA were prepared by applying the suspension mixed with SARS-CoV–infected Vero E6 cells from one patient (patient 4) and uninfected cells. Slides were dried and fixed in acetone. The conjugates used were goat antihuman IgG conjugated to fluorescein isothiocyanate (Organon Teknika-Cappel, Turnhout, Belgium). The starting dilution of serum specimens was 1:25 (*5*). Ten serum samples obtained from 10 pregnant women during routine prelabor check-ups were used as control sera. Two IVIG products, one domestic (from Taiwanese donors) and one imported (Bayer, Leverkusen, Germany), were also tested for the presence of antibody.

**Figure 2:**
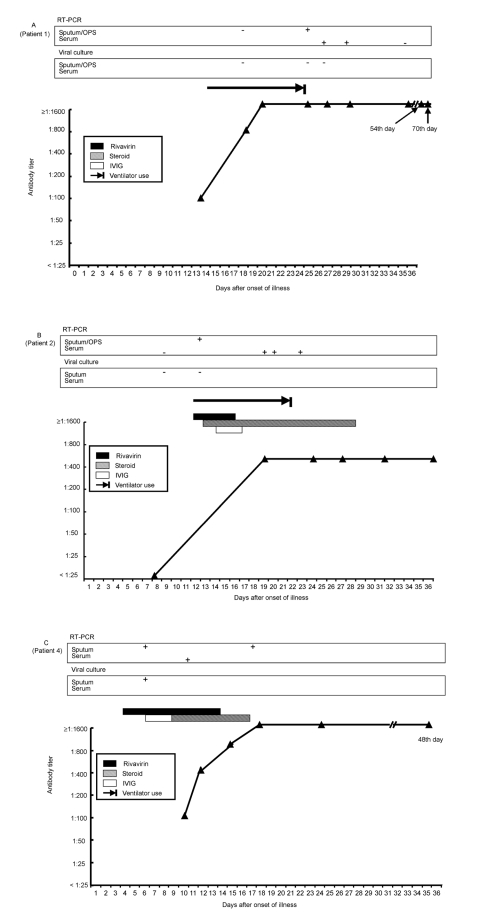
Timelines of positive reverse-transcription polymerase chain reaction, antibody responses and treatment regimens (ribavirin, corticosteroid, and intravenous immunoglobulin) after onset of disease in seven patients with severe acute respiratory syndrome. Panels A–C indicate patients 1, 2, and 4.

All serum samples from the 10 pregnant women and the two IVIG products were negative for IgG antibody (<1:25) to SARS-CoV. Six patients had detectable IgG antibody to SARS-CoV during the course of illness, and all of them had at least fourfold elevation of antibody levels in acute- and convalescent-phase serum samples (peak levels range 1:400– ≥1:1600) (Figure 2). Antibody titers (>1:25) of these six patients could be detected 9–18 days (mean 12.3 days) after the onset of illness. The antibody titer increased to a plateau 4–10 days after the appearance of antibody. The high antibody levels might last for 1 to >2 months after onset of illness (Figure 2). One previously healthy patient (patient 7) with positive SARS-CoV RNA by RT-PCR from both sputum and serum specimens had no detectable antibody to SARS-CoV in serum specimens obtained 7, 10, 14, and 24 days after illness onset. Although the antibody levels reached a plateau in all patients, viral RNA persisted in the serum samples from patients 1 and 2 and sputum from patients 1 and 4 for 19 to 29 days after onset of their illness.

Although four patients had received ribavirin, corticosteroid, and IVIG treatment in the early stage of the disease, antibody was detected as early as 10–12 days after the onset of illness. The peak level of antibody was 1:400 in patients 2 and 6, 1:800 in patient 3, and >1:1600 in patient 1.

## Conclusions

Serologic study indicated that the antibody to SARS-CoV appeared as early as 9 days after disease onset and that a high level of antibody could last for 1–2 months after disease onset. Previous reports indicated that the mean time for IgG seroconversion was 20 days and may start as early as 9–10 days. Our finding supported the results of Peiris et al. (*7*,*12*). Levels and appearance of antibody to SARS-CoV did not seem to be influenced by the use of ribavirin and immunosuppressive or immunomodulatory agents (corticosteroid or IVIG, a blood product prepared from the serum of 1,000 to 15,000 donors per batch) (*13*).

Third, the long-term persistence (19–29 days after illness onset) of viral RNA in the serum and sputum specimens of the SARS-CoV-specific IgG seroconverters is an important finding. Prolonged shedding of viral RNA in respiratory secretions (11 days after illness onset), plasma (up to 9 days), and stool specimens (25 days) was documented previously (*6*). Further studies are needed to determine whether the viable viral particles existed in body fluids in the presence of high antibody to the virus. Finally, one SARS patient, who did not have an underlying coexisting condition and did not receive any immunosuppressive agents during hospitalization, did not have detectable antibody to SARS-CoV 24 days (>21 days) after illness onset. The serum and sputum RT-PCR for SARS-CoV were positive in this patient, and the sequence was confirmed. Whether the patient was anergic to SARS-CoV infection is unknown. A later serum sample taken in the convalescent stage should be tested to determine whether this patient subsequently seroconverts (*7*).

The upsurge of IgG antibody to SARS-CoV and its correlation with the progression of ARDS, necessitating ventilator support in four of the seven patients, was evident. Previous study suggested that an overexuberant host response rather than uncontrolled viral replication, contributed to severe clinical symptoms and progressive lung damage (*12*). Whether the addition of SARS-CoV–specific antibody in SARS patients further aggravated the preexisting overactive immune-mediated deterioration was unclear.

High concentrations of viral RNA, up to 100 million molecules per milliliter, were detected in a sputum sample from an index patient on day 9 (*6*). In the present series, a physician contracted the infection from a patient (patient 2) 12 days after the onset of symptoms, indicating that shedding of the virus from the respiratory tract of symptomatic SARS patients may last for >12 days. Viral RNA in the sputum samples of patient 2 collected 12 days after the onset of symptoms supports this clinical finding.

Dual infection caused by *M. pneumoniae* and SARS-CoV was found in patient 5. No evidence of *M. pneumoniae* infection existed in patient 6 from the same cluster. This finding is similar to a previous report (*6*). Four of our patients had elevated IgG antibody titers for *C. pneumoniae,* and five had elevated antibody titers against parainfluenzavirus 1, 2, or 3 in acute-phase serum samples without a fourfold rise of titers in convalescent-phase serum samples. Whether the antibody responses of these patients reflected past infections from *C. pneumoniae,* parainfluenzavirus, or both, or merely a cross-reaction with antibody against SARS-CoV virus remains unclear.

As of May 16 2003, data of complete genomic sequences for 13 SARS-CoV strains isolated from Hong Kong, Singapore, China, Canada, Vietnam, and Taiwan were available in GenBank. The number of nucleotides ranged from 29,705 (SIN2677 strain) to 29,751 (TOR2) (*14*,*15*). Since February 2003, at least three different clusters of SARS outbreaks occurred in different parts of Taiwan, and five strains were identified from patients in these clusters. The availability of the sequence data of different strains in a given geographic area will have an immediate impact on the effort to trace the origins and transmission of SARS-CoV and develop novel rapid diagnostic tests and a vaccine.

In summary, analysis of these seven patients with virologically or serologically documented infections caused by SARS-CoV in Taiwan not only extended the knowledge of this emerging novel disease but also provided microbiologic and immunologic clues for the physicians caring for patients suspected of having this disorder. Viral RNA may persist for some time in patients who seroconvert, and some patients may lack an antibody response to SARS-CoV >21 days after illness onset. An upsurge of antibody response is associated with the aggravation of respiratory failure that required ventilator support.
